# Cross-sectional and longitudinal associations between arts engagement, loneliness, and social support in adolescence

**DOI:** 10.1007/s00127-022-02379-8

**Published:** 2022-11-07

**Authors:** Jessica K. Bone, Daisy Fancourt, Meg E. Fluharty, Elise Paul, Jill K. Sonke, Feifei Bu

**Affiliations:** 1 Research Department of Behavioural Science and Health, Institute of Epidemiology and Health Care, University College London, 1-19 Torrington Place, London, WC1E 7HB UK; 2 Center for Arts in Medicine, University of Florida, Gainesville, FL USA

**Keywords:** Loneliness, Social support, Arts, Culture, Extracurricular activities

## Abstract

**Purpose:**

Although arts engagement holds promise for reducing loneliness and enhancing social support, previous research has focussed on older adults. We investigated whether arts engagement was associated with loneliness and social support during adolescence.

**Methods:**

We included 11,780 adolescents aged 11–21 years from the National Longitudinal Study of Adolescent to Adult Health, a nationally representative cohort study. We measured whether adolescents engaged in school-based arts activities (band, book club, chorus, choir, cheerleading, dance, drama club, newspaper, orchestra) at wave one (1994–1995). Loneliness and perceived social support from peers were measured at waves one and two (1996). We used logistic regression to test whether arts engagement was associated with concurrent and subsequent loneliness and social support.

**Results:**

Arts engagement was not associated with concurrent or subsequent loneliness. Compared to not engaging, doing one or more school-based arts activities was associated with 59% higher odds of high social support concurrently (odds ratio [OR] = 1.59, 95% CI = 1.32–1.91). However, this cross-sectional association was attenuated after adjusting for demographic, socioeconomic, and health-related covariates (adjusted OR [AOR] = 1.16, 95% CI = 0.95–1.42). In contrast, doing arts activities was associated with 28% higher odds of reporting high social support one year later (AOR = 1.28, 95% CI = 1.03–1.59), independent of covariates and previous social support.

**Conclusions:**

Extracurricular arts activities are associated with increased odds of reporting good subsequent social support from peers. This may be because they provide opportunities for social engagement, developing friendships, and building a sense of community. Exploring these associations in more detail should be a priority, enabling better understanding of this strategy for enhancing social ties during adolescence.

**Supplementary Information:**

The online version contains supplementary material available at 10.1007/s00127-022-02379-8.

## Introduction

The importance of social relationships for health is widely recognised, with loneliness and social support a focus of research. Loneliness is a subjective negative feeling arising from a mismatch between a person’s desired level of meaningful social relationships and what they perceive they have [1]. It is distinct from, but strongly related to, perceived social support. Perceived social support refers to beliefs about the quantity and quality of support that is potentially available from a person’s relationships and social contacts [2]. Although they are related, social support offers a more positive framing than the problem-focussed language of loneliness and may provide an easier target for interventions to improve social outcomes [3]. Adolescents and young adults are at particular risk of feeling lonely and lacking social support [4, 5]. During adolescence, individuals spend increasing amounts of time with their peers and become more dependent on peers for social support [6, 7]. The quality of one’s social ties is therefore an important risk factor for the development of health problems in adolescence [7, 8]. Loneliness and lack of social support are associated with higher rates of depression and anxiety, suicidal ideation and attempts, and poorer general health both during adolescence and into adulthood [9–12].

Extracurricular arts activities (e.g., dance, music, and book clubs) present a potential strategy for reducing loneliness and enhancing social support during adolescence. The United Kingdom (UK) government’s strategy for reducing loneliness emphasised the importance of the arts and creativity to help people become more connected [13]. Arts activities may provide opportunities for safe social interactions and addressing maladaptive cognitions, which are the most effective elements of interventions targeting loneliness [14, 15]. In a large qualitative study of adults who engaged in the arts, 82% reported that arts engagement helped them feel connected to other people [16]. Arts engagement can also lead to increases in social wellbeing, as well as chances to form new relationships and social identities and increase self-esteem, empathy, and motivation [17, 18].

Although arts engagement holds promise for reducing loneliness and enhancing social support during adolescence, most research to date has focussed on older adults. Reviews have identified that participatory arts interventions can reduce loneliness and social isolation in older adults [19, 20]. For example, choir groups, making music, and arts and crafts programmes can decrease loneliness, facilitate new social relationships, and increase perceptions of closeness among participants [21–24]. More frequent receptive arts engagement (experiencing the arts as a listener, audience member, or visitor, e.g., going to a theatre or museum) is also associated with lower odds of loneliness, both cross-sectionally and ten years later [25]. Another observational study, which included adults of all ages but tested only cross-sectional associations, found that more receptive engagement was associated with less loneliness and more social support [26]. In contrast, participatory arts activities (actively creating and participating in the arts, e.g., painting or dancing) were not consistently associated with loneliness or social support [26].

To our knowledge, no studies have yet investigated whether engaging in extracurricular arts activities is associated with loneliness and social support during adolescence. There is some evidence that extracurricular activities help adolescents to develop new friendships, and arts activities (compared to sports or academic activities) may be most likely to lead to friendships [27]. For young adults, music, recreational dance, and theatre groups can promote social connectedness and improve peer relationships [28–31]. However, this evidence is mainly from small intervention studies that are prone to self-selection bias, often focus on specific diagnostic groups, only have short follow-up periods, and do not randomise participants or account for previous levels of social connectedness.

In this study, we aimed to investigate whether engagement in participatory extracurricular arts activities (band, book club, chorus/choir, cheerleading/dance, drama club, newspaper, and orchestra) was associated with concurrent and subsequent loneliness and social support from peers during adolescence. We included adolescents aged 11–21 years from the National Longitudinal Study of Adolescent to Adult Health (Add Health) [32]. We hypothesised that engagement in more extracurricular activities would be associated with lower odds of loneliness and higher odds of good social support, both concurrently and one year later.

## Methods

### Sample

Participants were drawn from Add Health; a longitudinal study of a nationally representative sample of adolescents who were in grades 7–12 (aged 12–18 years) in 1994–1995 and have been followed for five waves [32]. We included the restricted-use school, in-home, and parental data. Participants who completed waves one (1994–1995) and two (1996) of Add Health were eligible; arts engagement, loneliness, and social support were measured only in these waves. In total, 13,568 adolescents completed interviews at waves one and two, 11,923 of whom also had complete parental data at wave one. We included participants with complete data on loneliness and social support at waves one and two, resulting in a final sample of 11,780 adolescents.

### Ethical approval

All participants gave informed consent, and this study has Institutional Review Board approval from the University of Florida (IRB201901792) and ethical approval from University College London Research Ethics Committee (project 18,839/001).

### Arts engagement

Engagement in 33 school-based extracurricular activities was measured at wave one. From these questions, we selected all items that were related to the arts, which included seven activities. Adolescents were asked whether they were participating, or planned to participate, in any of the following clubs, organizations, or teams at school (yes, no): band; book club; chorus/choir; cheerleading/dance; drama club; newspaper; or orchestra. Using these items, we created a binary indicator of whether adolescents had participated in extracurricular arts activities (yes, no). In a sensitivity analysis, we attempted to quantify the magnitude of adolescents’ involvement in the arts by creating a sum score of the total number of extracurricular art types engaged in, with higher scores indicating more diverse engagement (range 0–7).

### Outcomes

*Loneliness* was measured at waves one and two with one question “How often was the following true during the past week? You felt lonely.” A binary indicator of loneliness was created by collapsing response options into not lonely (never or rarely) versus lonely (sometimes, a lot of the time, most or all of the time). This threshold was chosen as very few adolescents reported feeling lonely a lot of the time (5%) or most or all of the time (2%) at wave one.

*Social support* was measured at waves one and two with one question developed for Add Health. Adolescents were asked how much they felt that their friends cared about them. Five response options ranged from not at all to very much. As responses were skewed, a binary indicator of peer social support was created by collapsing response options into low (friends care not at all, very little, somewhat) versus high (friends care quite a bit, very much).

### Covariates

Previous research has shown that a range of demographic, socioeconomic, and health-related factors are associated with arts engagement, loneliness, and social support [33–35]. On average, younger males who are Black, Indigenous, and people of colour (BIPOC groups), have less education, have lower socioeconomic status, whose parents have less education, and who live in more deprived areas are less likely to participate in the arts [33–35]. In line with this evidence, we included sociodemographic and health-related factors measured at wave one as covariates. *Adolescent-reported covariates* were age (years), gender (male, female), race/ethnicity (White, Black/African American, Asian/Pacific Islander, Other [including Hispanic, American Indian/Native American, and Other]), first language spoken at home (English, non-English), and how often a health or emotional problem caused them to miss a social or recreational activity (never, a few times, once a week or more). *Parent-reported covariates* were highest level of parental education (less than high school, high school, some college, college graduate), parental marital status (married, unmarried [including divorced, separated, widowed, and never married]) and household income (quartiles: $0-$20,000, $21,000-$38,000, $39,000-$60,000, > $60,000). Finally, *interviewers reported* the urbanicity of adolescents’ home location (urban, suburban, rural).

### Statistical analyses

All analyses were performed using Stata 16 [36] and we report and interpret our findings using the STROBE guidelines (Table S1) [37]. We first investigated whether arts engagement was associated with concurrent loneliness and social support. We tested the association between arts engagement and loneliness measured simultaneously at wave one using logistic regression. We then repeated these analyses for our second outcome, social support. Models are presented before and after adjustment for covariates.

Next, we tested whether arts engagement was associated with subsequent loneliness and social support, measured at wave two. We first tested whether arts engagement at wave one was associated with loneliness at wave two using logistic regression, then adjusted this model for covariates, and finally additionally adjusted for loneliness at wave one. Adjusting longitudinal models for loneliness measured at wave one considers that loneliness at follow-up is not only related to arts engagement, but also to previous loneliness. The fully adjusted model thus estimates the association between arts engagement and change in loneliness one year later. We then repeated this approach for social support. All analyses were weighted to account for the complex survey design, non-response, and clustering of participants within schools.

For participants with missing data on arts engagement or covariates, we imputed data using multiple imputation by chained equations (MICE) [38]. We used linear, logistic, ordinal, and multinomial regression according to variable type, generating 30 imputed data sets (maximum missing data 24%; Table S2). The imputation model included all variables used in analyses, auxiliary variables (grade, general health rating), and survey weights. All variables were successfully imputed. The results of analyses did not vary between complete cases and imputed data sets (Tables S3–S5), so imputed findings are reported.

### Sensitivity analyses

To ensure our binary outcomes did not obscure associations between arts engagement and frequency of feeling lonely, we tested the association between arts engagement and loneliness frequency (never or rarely, sometimes, a lot of the time, most or all of the time) using ordinal logistic regression. We then repeated this analysis for the level of peer social support (not at all, very little, somewhat, quite a bit, very much).

Given that we used a binary indicator of arts engagement, we performed a sensitivity analysis quantifying the magnitude of adolescents’ involvement in the arts. We tested whether the number of different types of extracurricular arts activities engaged in (0–7) was associated with loneliness and social support concurrently and longitudinally.

Finally, we investigated whether the associations between engaging in extracurricular arts activities, loneliness, and social support held after adjusting for engagement in other (non-arts) extracurricular activities. We created a binary variable indicating engagement in any of the 26 other extracurricular activities measured, comprising language (e.g., French), academic (e.g., history), and sports clubs (e.g., football), and other clubs/societies (e.g., student council). For each sensitivity analysis, we generated another 30 imputed data sets including all relevant variables with MICE.

## Results

After weighting of the 11,780 participants, 50% were female and 74% were White, whereas 15% were Black/African American, 3% were Asian/Pacific Islander, and 8% were of Other races/ethnicities (Table 1). The mean age was 15.02 years (standard deviation [SD] = 1.62, range = 11–21) at wave one, and 15.89 years (SD = 1.64, range = 11–23) at wave two. Overall, 36% of adolescents participated in an extracurricular arts activity. At wave one, 34% of adolescents felt lonely (sometimes, a lot of the time, or most or all the time), and this remained similar at wave two (33%). At waves one and two, 85% and 86% of participants reported high levels of social support (friends care quite a bit or very much).

**Table 1:** Sociodemographic characteristics of the sample at wave one

	Overall sample
Mean (SD)	
Age (years)	15.02 (1.62)
Percentage	
Gender	
Male	50%
Female	50%
Race/ethnicity	
White	74%
Black/African American	15%
Asian/Pacific Islander	3%
Other	8%
First language	
English	94%
Non-English	6%
Urbanicity	
Urban	32%
Suburban	39%
Rural	29%
Missed activity due to health	
Never	75%
A few times	23%
Once a week or more	2%
Parental education	
Less than high school	16%
High school	33%
Some college	29%
College graduate	22%
Household income (quartiles)	
$0-$20,000	26%
$21,000-$38,000	26%
$39,000-$60,000	28%
$61,000 +	20%
Parental marital status	
Married	72%
Unmarried	28%

*N* = 11,780. Results weighted and based on 30 multiply imputed data sets. *SD* standard deviation

### Concurrent associations

There was no evidence that arts engagement was associated with concurrent loneliness (Table 2). In contrast, arts engagement was associated with 59% higher odds of reporting high concurrent social support compared to not engaging (odds ratio [OR] = 1.59, 95% confidence interval [CI] = 1.32–1.91; Table 3). However, after adjusting for covariates (age, gender, race/ethnicity, first language, urbanicity, missed activity due to health, parental education, parental marital status, and household income), the evidence for this association was attenuated (adjusted OR [AOR] = 1.16, 95% CI = 0.95–1.42).

**Table 2:** Logistic regression models testing associations between extracurricular arts engagement (yes, no) and concurrent and subsequent loneliness

Loneliness	Odds ratio	95% CI	*p* value
Concurrent			
Unadjusted	0.98	0.86–1.12	0.759
Adjusted^a^	0.90	0.78–1.05	0.189
Longitudinal			
Unadjusted	1.00	0.87–1.13	0.946
Adjusted^a^	0.97	0.83–1.12	0.640
Additionally adjusted^b^	0.99	0.85–1.16	0.938

*n* = 11,780. Results weighted and based on 30 multiply imputed data sets. 95% CI: 95% confidence interval

^a^Models adjusted for age, gender, race/ethnicity, first language, urbanicity, missed activity due to health, parental education, parental marital status, and household income

^b^Model additionally adjusted for loneliness at wave one

**Table 3:** Logistic regression models testing associations between extracurricular arts engagement (yes, no) and concurrent and subsequent high social support

Social support	Odds ratio	95% CI	*p* value
Concurrent			
Unadjusted	1.59	1.32–1.91	< 0.001
Adjusted^a^	1.16	0.95–1.42	0.151
Longitudinal			
Unadjusted	1.76	1.45–2.13	< 0.001
Adjusted^a^	1.31	1.06–1.61	0.013
Additionally adjusted^b^	1.28	1.03–1.59	0.024

*n* = 11,780. Results weighted and based on 30 multiply imputed data sets. 95% CI: 95% confidence interval

^a^Models adjusted for age, gender, race/ethnicity, first language, urbanicity, missed activity due to health, parental education, parental marital status, and household income

^b^Model additionally adjusted for social support at wave one

### Longitudinal associations

As in concurrent models, arts engagement was not associated with subsequent loneliness (Table 2). However, compared to not engaging, doing one or more arts activity was associated with 76% higher odds of high social support (OR = 1.76, 95% CI = 1.45–2.13; Table 3). Although attenuated, there was still evidence for this association after adjustment for covariates (AOR = 1.31, 95% CI = 1.06–1.61). After additionally adjusting for previous social support, arts engagement was associated with 28% higher odds of reporting high social support one year later (AOR = 1.28, 95% CI = 1.03–1.59).

### Sensitivity analyses

At wave one, 66% of adolescents never or rarely felt lonely, 27% sometimes, 5% a lot of the time, and 2% felt lonely most or all of the time. Arts engagement was not associated with concurrent or subsequent frequency of feeling lonely, before or after adjusting for covariates and previous loneliness (Table S6).

Also at wave one, 43% of adolescents felt that their friends cared for them very much, 42% quite a bit, 12% somewhat, 2% very little, and 1% not at all. Concurrently, arts engagement was associated with higher odds of reporting high levels of social support, before and after adjustment for covariates (Table S7). Arts engagement was also longitudinally associated with higher levels of social support one year later, before and after adjustment for covariates and previous social support.

Quantifying the magnitude of arts involvement, we found that on average adolescents did 0.50 (SD = 0.80, range = 0–7) different extracurricular arts activities. As in the main analyses, there was no evidence that arts engagement was associated with concurrent or subsequent loneliness (Table S8). For social support, each additional type of arts activity engaged in was associated with greater odds of reporting high concurrent social support, but this was attenuated after adjustment for covariates (Table S9). Similarly, there was only evidence for the longitudinal association before adjustment for covariates and previous social support.

Non-arts extracurricular activities were done by 70% of adolescents. Engaging in non-arts (but not arts) extracurricular activities was associated with lower odds of concurrent and subsequent loneliness (Table S10). In contrast, engaging in both arts and non-arts extracurricular activities was associated with subsequent social support (Table S11). Associations between arts engagement and social support were very similar to the main analyses despite also including non-arts extracurricular activities in the same model.

## Discussion

Using data from a large longitudinal study, we explored whether engagement in extracurricular arts activities was associated with loneliness and social support during adolescence. We found no evidence that engagement (in band, book club, chorus/choir, cheerleading/dance, drama club, newspaper, or orchestra) was associated with loneliness. Concurrently, engaging in one or more of these activities was associated with higher odds of reporting good peer social support, but this evidence was attenuated after adjusting for sociodemographic and health-related covariates. However, in a sensitivity analysis, arts engagement was associated with higher levels of social support even after adjustment for covariates. Additionally, doing extracurricular arts activities was associated with higher odds of good social support one year later, independent of covariates and previous social support. These findings were robust to a range of sensitivity analyses, including accounting for engagement in other (non-arts) extracurricular activities. This suggests that arts engagement is associated with higher perceived social support from peers, regardless of whether adolescents are doing other extracurricular activities.

The lack of association with loneliness contrasts with previous evidence that participatory arts interventions reduce loneliness in older adults [19, 20]. This could be because of differences in the causes and subjective experience of loneliness between older adults and adolescents [5]. Most adolescents spend much of their time at school, meaning factors such as school environment may have a larger influence on loneliness than extracurricular arts activities [39]. Additionally, the type of extracurricular activities that adolescents participate in may relate to their social standing at school. The difference to previous findings in older adults could also be due to limitations of the loneliness measure in Add Health. Although assessing loneliness with a single item has been recommended [13], it may not have been sufficient to detect associations with arts engagement. Loneliness is often divided into emotional and social loneliness. Emotional loneliness results from the lack of a close emotional attachment or intimate relationship (e.g., a partner or best friend), whereas social loneliness results from the lack of an engaging social network or broader group (e.g., friends and peers) [40]. As arts activities are more likely to provide access to a group of peers than a partner or best friend, they may only reduce social loneliness, and not emotional loneliness. In adults, participatory arts engagement was associated with lower social loneliness but higher emotional loneliness [26]. This may not be apparent when using a measure that does not distinguish between these two subtypes of loneliness. In our sensitivity analysis, doing non-arts extracurricular activities was associated with less loneliness. Why participation in a broad range of clubs, but not arts activities, is associated with loneliness requires further investigation. Given that receptive arts engagement is more strongly associated with loneliness than participatory engagement in adults [25, 26], future research should also explore whether receptive arts activities can reduce loneliness in young people.

Doing extracurricular arts activities was associated with higher perceived social support concurrently and one year later, although the cross-sectional association was not independent of covariates. Despite this, in sensitivity analyses, arts engagement was associated with higher levels of social support, even after adjustment for covariates. It is therefore possible that the binary indicator of low versus high social support was not sensitive enough to detect the concurrent associations after accounting for the wide range of covariates. In further sensitivity analyses, both arts and non-arts extracurricular activities were associated with subsequent social support, although the evidence was stronger for non-arts activities. Most adolescents participated in at least one extracurricular activity, so it is possible that not participating in any activities (whether artistic or not) is particularly detrimental. Our findings contrast with previous research, which suggested that arts activities are more likely to lead to new friendships than sports or academic clubs because they are less competitive and may appeal to a lower status group, allowing stronger friendships to form [27].

There are a range of mechanisms through which extracurricular arts activities could increase social support. They provide opportunities to connect with others, improve social relationships, develop social skills, and build a sense of community and shared purpose [17]. Participatory activities such as book clubs, singing, and dance make adults feel connected to others [16]. In young adults, music, dance, and theatre group interventions promote social connectedness and improve relationships [28–31]. Given that arts engagement was associated with subsequent social support, encouraging adolescents to participate in school-based extracurricular arts activities may be important. Further intervention studies are needed to explore whether arts activities can causally change social support over time.

This study has several strengths. We used data from a large longitudinal study with rich data on covariates, meaning we could adjust for a range of sociodemographic and health-related factors. We included two distinct outcomes, assessing different aspects of social networks. Future research could extend this work by considering further social outcomes that affect health, including social influence and access to resources. Additionally, in our analyses, we clustered participants within schools, accounting for the fact that adolescents within schools are more like each other than adolescents at other schools.

However, this study also has some limitations. The measures of art engagement, loneliness, and social support in Add Health could have been more detailed. These measures were developed for Add Health, but their validity has not been established. We could not distinguish adolescents who were currently participating in extracurricular activities from those who planned to participate. We used a binary indicator of whether adolescents participated (or planned to participate) in extracurricular arts activities because we did not have information on the number of activities done within each type of arts engagement or the frequency of engagement. It is likely that different types of arts engagement included different levels of commitment, and someone who dedicated substantial time to one activity would have been ranked lower than an individual who dedicated small amounts of time to multiple activities, even though their overall time commitment may have been higher. In line with these limitations, sensitivity analyses assessing the number of different types of arts activities adolescents engaged in provided less clear evidence for the association with social support. Additionally, as these measures were only included in waves one and two, we could not use more sophisticated analytic approaches. Future research should investigate the developmental trajectories of arts engagement, loneliness, and social support in more detail.

Although Add Health included a nationally representative sample of the target population (1994–1995 grades 7–11), this sample is no longer representative of the US population, meaning our findings may not be generalisable to all adolescents. Generational differences in social and cultural context, such as the use of social media, may also mean the factors associated with loneliness and social support in adolescence have changed since the 1990s. Our findings should thus be replicated with more recent data, although this is challenging due to the lack of current data on arts and cultural engagement in representative cohorts. Furthermore, there is a social gradient in extracurricular arts engagement, loneliness, and social support [34, 41, 42]. Despite adjusting for sociodemographic and health-related factors, it remains possible that our results are due to self-selection or residual confounding. The longitudinal association could be a result of reverse causation, with higher levels of social support leading participants to engage in more activities, although the lack of cross-sectional evidence after adjusting for covariates suggests that this is not the case. We recognize that we used an overly simple race/ethnicity variable (White, Black, Asian/Pacific Islander, Other) due to small numbers in non-White groups. This approach conflates experiences across diverse racial/ethnic groups, which might be particularly problematic as these groups may not have equal access to arts resources [33]. Future research must use more diverse samples and collect detailed data on race/ethnicity, while considering the potential influence of structural racism on loneliness and perceived social support.

Overall, we found no evidence that engagement in extracurricular arts activities was associated with loneliness during adolescence. In contrast, engagement in more extracurricular arts activities was associated with higher odds of good social support from peers, both concurrently and one year later, independent of participation in other extracurricular activities. Given that adolescents are at particular risk of feeling lonely and lacking social support, and loneliness and lack of social support are associated with a range of negative outcomes, identifying ways to reduce loneliness and enhance social support at a population level is particularly important. Despite the lack of consistent evidence for the effectiveness of extracurricular arts activities in this study, there are well-established benefits of arts engagement for health, with evidence that these may benefits may occur through social processes [17]. A key priority should thus be to explore the associations between arts engagement, loneliness, and social support in more detail, enabling better understanding of this potential strategy for reducing loneliness and enhancing social support during adolescence.

## Supplementary Information

Below is the link to the electronic supplementary material.

Supplementary file1 (PDF 280 KB)

## Data Availability

The data that support the findings of this study are available from Add Health but restrictions apply to the availability of these data, which were used under license for the current study, and so are not publicly available. Those interested in obtaining restricted-use data files from Add Health should contact Add Health, The University of North Carolina at Chapel Hill, Carolina Population Center, Carolina Square, Suite 210, 123 W. Franklin Street, Chapel Hill, NC 27516 (addhealth_contracts@unc.edu).
