# Supplementary material for: Cross-sectional and longitudinal associations between arts engagement, loneliness, and social support in adolescence
Source: Soc Psychiatry Psychiatr Epidemiol. 2022 Nov 7;58(6):931–8. doi: 10.1007/s00127-022-02379-8 (PMC10241709; doi:10.1007/s00127-022-02379-8)
Supplement: Supplementary file 1 — Supplementary file1 (PDF 280 KB) [file 127_2022_2379_MOESM1_ESM.pdf]

Supplementary Materials

Table S1. *STROBE Statement: Checklist of items that should be included in reports of cohort studies.*

Item	No.	Recommendation	Page
Title and abstract	1	(*a*) Indicate the study's design with a commonly used term in the title or the abstract	1
		(*b*) Provide in the abstract an informative and balanced summary of what was done and what was found	1
**Introduction**			
Background/rationale	2	Explain the scientific background and rationale for the investigation being reported	1-2
Objectives	3	State specific objectives, including any prespecified hypotheses	2
**Methods**			
Study design	4	Present key elements of study design early in the paper	2
Setting	5	Describe the setting, locations, and relevant dates, including periods of recruitment, exposure, follow-up, and data collection	2
Participants	6	(*a*) Give the eligibility criteria, and the sources and methods of selection of participants. Describe methods of follow-up	2
		(*b*) For matched studies, give matching criteria and number of exposed and unexposed	N/A
Variables	7	Clearly define all outcomes, exposures, predictors, potential confounders, and effect modifiers. Give diagnostic criteria, if applicable	2-3
Data sources/ measurement	8	For each variable of interest, give sources of data and details of methods of assessment (measurement). Describe comparability of assessment methods if there is more than one group	2-3
Bias	9	Describe any efforts to address potential sources of bias	3-4
Study size	10	Explain how the study size was arrived at	2-3
Quantitative variables	11	Explain how quantitative variables were handled in the analyses. If applicable, describe which groupings were chosen and why	2-3
Statistical methods	12	(*a*) Describe all statistical methods, including those used to control for confounding	3-4
		(*b*) Describe any methods used to examine subgroups and interactions	N/A
		(*c*) Explain how missing data were addressed	3-4
		(*d*) If applicable, explain how loss to follow-up was addressed	N/A
		(*e*) Describe any sensitivity analyses	3-4
**Results**			
Participants	13	(a) Report numbers of individuals at each stage of study—eg numbers potentially eligible, examined for eligibility, confirmed eligible, included in the study, completing follow-up, and analysed	2-3
		(b) Give reasons for non-participation at each stage	2-3
		(c) Consider use of a flow diagram	N/A
Descriptive data	14	(a) Give characteristics of study participants (eg demographic, clinical, social) and information on exposures and potential confounders	4
		(b) Indicate number of participants with missing data for each variable of interest	Table S1
		(c) Summarise follow-up time (eg, average and total amount)	N/A
Outcome data	15	Report numbers of outcome events or summary measures over time	4
Main results	16	(*a*) Give unadjusted estimates and, if applicable, confounder-adjusted estimates and their precision (eg, 95% confidence interval). Make clear which confounders were adjusted for and why they were included	4-5 Tables 2-3 4
		(*b*) Report category boundaries when continuous variables were categorized	4

		(*c*) If relevant, consider translating estimates of relative risk into absolute risk for a meaningful time period	N/A
Other analyses	17	Report other analyses done—eg analyses of subgroups and interactions, and sensitivity analyses	5
**Discussion**			
Key results	18	Summarise key results with reference to study objectives	5
Limitations	19	Discuss limitations of the study, taking into account sources of potential bias or imprecision. Discuss both direction and magnitude of any potential bias	7-8
Interpretation	20	Give a cautious overall interpretation of results considering objectives, limitations, multiplicity of analyses, results from similar studies, and other relevant evidence	5-8
Generalisability	21	Discuss the generalisability (external validity) of the study results	7
**Other information**			
Funding	22	Give the source of funding and the role of the funders for the present study and, if applicable, for the original study on which the present article is based	End

Table S2. *Missing data in arts engagement and covariates at wave one for the whole sample (n=11,780).*

	**Missing data**	
	N	%
Arts engagement	2773	23.54%
Age	3	0.03%
Gender	0	-
Race/ethnicity	3	0.03%
First language	0	-
Urbanicity	194	1.65%
Missed activity due to health	8	0.07%
Parental education	90	0.76%
Household income (quartiles)	1486	12.61%
Parental marital status	32	0.27%

Note. The sample was limited to participants no missing data in outcome measures at wave one or two.

Table S3. *Characteristics of the sample at wave one for complete cases only.*

	**Complete case sample**
**Mean (SD)**	
Age (years)	14.94 (1.62)
**Proportion**	
Gender	
Male	50%
Female	50%
Race/ethnicity	
White	76%
Black/African American	15%
Asian/Pacific Islander	3%
Other	6%
First language	
English	95%
Non-English	5%
Urbanicity	
Urban	31%
Suburban	37%
Rural	32%
Missed activity due to health	
Never	75%
A few times	23%
Once a week or more	2%
Parental education	
Less than high school	13%
High school	33%
Some college	31%
College graduate	23%
Household income (quartiles)	
$0-$20,000	23%
$21,000-$38,000	26%
$39,000-$60,000	30%
$61,000+	21%
Parental marital status	
Married	73%
Unmarried	27%
Engaged in the arts	37%
Lonely	33%
High social support	86%

Note. n=7,713. Results are weighted. SD: standard deviation.

Table S4. *Logistic regression models testing associations between extracurricular arts engagement and concurrent and subsequent loneliness for complete cases only.*

Loneliness	Odds ratio	95% CI	p value
**Concurrent**			
Unadjusted	0.98	0.85-1.13	0.771
Adjusted[a]	0.90	0.76-1.07	0.228
**Longitudinal**			
Unadjusted	1.02	0.90-1.17	0.744
Adjusted[a]	1.01	0.87-1.17	0.940
Additionally adjusted[b]	1.04	0.89-1.22	0.591

Note. n=7,713. Results are weighted. 95% CI: 95% confidence interval. a: models adjusted for age, gender, race/ethnicity, first language, urbanicity, missed activity due to health, parental education, parental marital status, and household income. b: model additionally adjusted for loneliness at wave one.

Table S5. *Logistic regression models testing associations between extracurricular arts engagement and concurrent and subsequent high social support for complete cases only.*

Social support	Odds ratio	95% CI	p value
**Concurrent**			
Unadjusted	1.60	1.31-1.96	<0.001
Adjusted[a]	1.16	0.93-1.45	0.174
**Longitudinal**			
Unadjusted	1.76	1.45-2.15	<0.001
Adjusted[a]	1.36	1.10-1.69	0.006
Additionally adjusted[b]	1.34	1.06-1.69	0.016

Note. n=7,713. Results are weighted. 95% CI: 95% confidence interval. a: models adjusted for age, gender, race/ethnicity, first language, urbanicity, missed activity due to health, parental education, parental marital status, and household income. b: model additionally adjusted for social support at wave one.

Table S6. *Ordinal logistic regression models testing associations between extracurricular arts engagement and concurrent and subsequent loneliness frequency.*

Loneliness frequency	Odds ratio	95% CI	p value
**Concurrent**			
Unadjusted	0.98	0.86-1.12	0.745
Adjusted[a]	0.90	0.77-1.05	0.187
**Longitudinal**			
Unadjusted	0.99	0.87-1.13	0.885
Adjusted[a]	0.96	0.83-1.11	0.577
Additionally adjusted[b]	0.99	0.85-1.15	0.895

Note. n=11,780. Results weighted and based on 30 multiply imputed data sets. 95% CI: 95% confidence interval. a: models adjusted for age, gender, race/ethnicity, first language, urbanicity, missed activity due to health, parental education, parental marital status, and household income. b: model additionally adjusted for loneliness frequency at wave one.

Table S7. *Ordinal logistic regression models testing associations between extracurricular engagement and concurrent and subsequent level of social support.*

Level of social support	Odds ratio	95% CI	p value
**Concurrent**			
Unadjusted	1.43	1.29-1.59	<0.001
Adjusted[a]	1.13	1.00-1.27	0.049
**Longitudinal**			
Unadjusted	1.51	1.36-1.69	<0.001
Adjusted[a]	1.17	1.03-1.32	0.015
Additionally adjusted[b]	1.14	1.00-1.29	0.043

Note. n=11,780. Results weighted and based on 30 multiply imputed data sets. 95% CI: 95% confidence interval. a: models adjusted for age, gender, race/ethnicity, first language, urbanicity, missed activity due to health, parental education, parental marital status, and household income. b: model additionally adjusted for level of social support at wave one.

Table S8. *Logistic regression models testing associations between the number of extracurricular arts activities engaged in and concurrent and subsequent loneliness.*

Loneliness	Odds ratio	95% CI	p value
**Concurrent**			
Unadjusted	0.99	0.92-1.07	0.773
Adjusted[a]	0.94	0.86-1.02	0.157
**Longitudinal**			
Unadjusted	1.00	0.93-1.07	0.995
Adjusted[a]	0.98	0.91-1.06	0.568
Additionally adjusted[b]	1.00	0.92-1.08	0.909

Note. n=11,780. Results are weighted and based on 30 multiply imputed data sets. 95% CI: 95% confidence interval. a: models adjusted for age, gender, race/ethnicity, first language, urbanicity, missed activity due to health, parental education, parental marital status, and household income. b: model additionally adjusted for loneliness at wave one.

Table S9. *Logistic regression models testing associations between the number of extracurricular arts activities engaged in and concurrent and subsequent high social support.*

Social support	Odds ratio	95% CI	p value
**Concurrent**			
Unadjusted	1.35	1.19-1.52	<0.001
Adjusted[a]	1.12	0.99-1.26	0.068
**Longitudinal**			
Unadjusted	1.35	1.18-1.55	<0.001
Adjusted[a]	1.12	0.98-1.28	0.083
Additionally adjusted[b]	1.10	0.96-1.26	0.176

Note. n=11,780. Results are weighted and based on 30 multiply imputed data sets. 95% CI: 95% confidence interval. a: models adjusted for age, gender, race/ethnicity, first language, urbanicity, missed activity due to health, parental education, parental marital status, and household income. b: model additionally adjusted for social support at wave one.

Table S10. *Logistic regression models testing associations between whether participants engaged in extracurricular arts activities, other (non-arts) extracurricular activities, and concurrent and subsequent loneliness.*

	Arts activities			Non-arts activities		
**Loneliness**	Odds ratio	95% CI	p value	Odds ratio	95% CI	p value
**Concurrent**						
Unadjusted	1.02	0.89-1.17	0.735	0.74	0.65-0.86	<0.001
Adjusted[a]	0.94	0.81-1.10	0.419	0.76	0.66-0.88	0.001
**Longitudinal**						
Unadjusted	1.04	0.91-1.18	0.591	0.76	0.67-0.87	<0.001
Adjusted[a]	0.99	0.86-1.16	0.946	0.81	0.70-0.92	0.002
Additionally adjusted[b]	1.02	0.87-1.18	0.841	0.86	0.74-0.99	0.042

Note. n=11,780. Results are weighted and based on 30 multiply imputed data sets. 95% CI: 95% confidence interval. a: models adjusted for age, gender, race/ethnicity, first language, urbanicity, missed activity due to health, parental education, parental marital status, and household income. b: model additionally adjusted for loneliness at wave one.

Table S11. *Logistic regression models testing associations between whether participants engaged in extracurricular arts activities, other (non-arts) extracurricular activities, and concurrent and subsequent high social support.*

	Arts activities			Non-arts activities		
**Social support**	Odds ratio	95% CI	p value	Odds ratio	95% CI	p value
**Concurrent**						
Unadjusted	1.50	1.25-1.81	<0.001	1.46	1.21-1.77	<0.001
Adjusted[a]	1.11	0.90-1.36	0.330	1.39	1.14-1.71	0.002
**Longitudinal**						
Unadjusted	1.66	1.37-2.02	<0.001	1.47	1.23-1.75	<0.001
Adjusted[a]	1.25	1.01-1.55	0.037	1.36	1.14-1.62	0.001
Additionally adjusted[b]	1.24	1.00-1.54	0.052	1.27	1.06-1.53	0.012

Note. n=11,780. Results are weighted and based on 30 multiply imputed data sets. 95% CI: 95% confidence interval. a: models adjusted for age, gender, race/ethnicity, first language, urbanicity, missed activity due to health, parental education, parental marital status, and household income. b: model additionally adjusted for social support at wave one.
